# Circadian Rhythms Coordinated With Gut Microbiota Partially Account for Individual Differences in Hepatitis B-Related Cirrhosis

**DOI:** 10.3389/fcimb.2022.936815

**Published:** 2022-07-01

**Authors:** Tongyao Wang, Xingyu Rong, Chao Zhao

**Affiliations:** ^1^ Ministry of Education (MOE)/National Health Commission (NHC)/Chinese Academy of Medical Science (CAMS) Key Lab of Medical Molecular Virology, School of Basic Medical Sciences & National Clinical Research Center for Aging and Medicine, Huashan Hospital, Shanghai Medical College, Fudan University, Shanghai, China; ^2^ Department of Medical Chemistry, Graduate School of Medicine, Kyoto University, Kyoto, Japan; ^3^ Shanghai Frontiers Science Center of Pathogenic Microbes and Infection, Shanghai, China

**Keywords:** circadian rhythms, cirrhosis, hepatitis B, gut microbiota, bile acids, immunomodulatory metabolites, metabolism, immune

## Abstract

Cirrhosis is the end stage of chronic liver diseases like chronic hepatitis B. In China, hepatitis B accounts for around 60% of cases of cirrhosis. So far, clinical and laboratory indexes for the early diagnosis of cirrhosis are far from satisfactory. Nevertheless, there haven’t been specific drugs for cirrhosis. Thus, it is quite necessary to uncover more specific factors which play their roles in cirrhosis and figure out the possible therapeutic targets. Among emerging factors taking part in the initiation and progression of cirrhosis, gut microbiota might be a pivot of systemic factors like metabolism and immune and different organs like gut and liver. Discovery of detailed molecular mechanism in gut microbiota and gut liver axis leads to a more promising prospect of developing new drugs intervening in these pathways. Time-based medication regimen has been proofed to be helpful in hormonotherapy, especially in the use of glucocorticoid. Thus, circadian rhythms, though haven’t been strongly linked to hepatitis B and its complications, are still pivotal to various pathophysiological progresses. Gut microbiota as a potential effective factor of circadian rhythms has also received increasing attentions. Here, our work, restricting cirrhosis to the post-hepatitis B one, is aimed to summarize how circadian rhythms and hepatitis B-related cirrhosis can intersect *via* gut microbiota, and to throw new insights on the development of new and time-based therapies for hepatitis B-related cirrhosis and other cirrhosis.

## Introduction

Cirrhosis is a kind of diffuse liver disease that progresses chronically. It is basically characterized with the degeneration and necrosis of hepatocytes, global fibrosis in liver, and the formation of regenerative nodules of hepatocytes. Thus, the hepatic lobules and blood vessels can be reconstructed, which consequently leads to hepatic insufficiency and severe clinical outcomes. Around 60% of cases of cirrhosis in China are related to HBV infection (Wang et al., 2014). A considerable proportion of hepatocellular carcinoma (HCC) cases develop on the basis of cirrhosis, which is consistent with the fact that about 80% of patients with HCC in China tested positive for HBsAg (Wang et al., 2014). Hepatic encephalopathy (HE) can also result from cirrhosis induced by hepatitis B and other non-viral hepatitis. Thus, more comprehensive and specific studies are still required for early diagnosis and more effective treatment. However, the huge difference in the outcome of hepatitis B carriers is still unclear. In this complex disease with long-term persistent infection, the integration of internal and external factors may be the root cause of the diversity of outcomes.

Among these potential causes, the regulation of biological rhythm may be a good mechanism to reconcile the different outcomes caused by internal and external factors. Light/dark cycles on the earth shaped the circadian clock in animals and plants. The circadian clock helps coordinate the vital activities with the existence of sunlight, which is the source of all energy on the earth. It is obvious that all cells in human body have a clock system (Rijo-Ferreira and Takahashi, 2019) or are regulated by suprachiasmatic nucleus (SCN), which is believed to transduce the photic cues to rhythmic physiological signals and thus to synchronize the physiological activity with the diurnal variations (Maury et al., 2010; Peek et al., 2015). In addition to the direct regulation of light through the central circadian clock and the intrinsic circadian clock system, behaviors demonstrating a rhythmic variation like diet (Chaix et al., 2019) and sleep (Huang et al., 2011) also play important roles in maintaining the homeostasis.

In particular, microbiota is an important factor that interacts with the biological rhythm of the body, and mediates internal and external factors. Gut microbiota has been shown to take part in the regulation and development of various functional systems and has exhibited complicated effects in the past decades. Gut microbiota consists of organisms from viruses and prokaryotes like archaebacteria and bacteria to eukaryotes like protists and fungi. These organisms together make up an acquired symbiotic part of human body and serve as one of the organs through their own structural components and metabolites, interacting with multiple systems (Donaldson et al., 2016). Since the gut microbiota is mainly located in gastrointestinal tract and has no access to the photic cues, diet cues play important roles in modulating the abundance and composition of the gut microbiota throughout one day. The interaction between gut microbiota and the host’s metabolic and immune system has been reported to be closely related to metabolic syndromes, autoimmune diseases, psychological illnesses, infectious diseases, and tumors (Cryan and Dinan, 2012; Guinane and Cotter, 2013). Hence, the gut microbiota probably mediates the impact of circadian rhythm on the immune and metabolic system and furtherly contributes to the development of various diseases.

**Figure 1:**
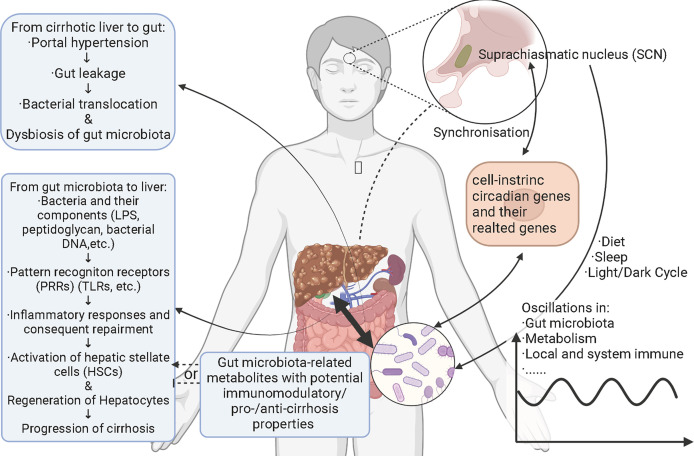
Schematic figure for interactions among hepatitis B-related cirrhosis, gut microbiota, and circadian rhythms. Circadian rhythms cause the oscillations and alterations of gut microbiota through diet, sleep, and light/dark cycles. Metabolism and local and systemic immune also oscillate with gut microbiota and both photic and nonphotic Zeitgebers. Gut microbiota impact the signaling pathways related to cell-intrinsic circadian clock *via* bacterial components and/or metabolites. Cell-intrinsic circadian clock synchronizes with the central circadian rhythm generated by suprachiasmatic nucleus. Hepatitis B-related cirrhosis leads to gut leakage mainly through portal hypertension. With the permeability of gut increasing, gut bacteria translocate to mesenteric lymph nodes and other parts of body, called bacterial translocation. At the same time, gut bacteria and bacterial components like lipopolysaccharides, peptidoglycan and bacterial DNAs translocate to liver *via* portal veins. These components activate pattern recognition receptors in liver and trigger inflammatory responses, followed by repairment. Hepatic stellate cells are consequently activated and the generation of hepatocytes starts. Finally, cirrhosis is aggravated. Gut microbiota-derivate metabolites with immunomodulatory/pro-/anti-cirrhosis properties may also affect the progression of cirrhosis. Relationships between cirrhosis and circadian rhythms and the underlying mechanisms remain elusive.

A large gap still exists between circadian rhythms and liver cirrhosis with the basis of hepatitis B. Different outcomes of chronic hepatitis B and its complications are also bothering researchers and clinical physicians. Besides, the optimization of treatments for hepatitis B-related cirrhosis and corresponding theoretical basis need further exploration. As relatively individualized factors, gut microbiota may coordinate with circadian rhythms to influence the progression of hepatitis B- related cirrhosis and to provide new therapeutical targets. Here this review is aimed to conclude how gut microbiota take part in the initiation and progression of hepatitis-B related cirrhosis in metabolic and immune ways as well as the potential manner in which circadian rhythms may play a role in shaping the systematic and liver local microecology (**Figure 1**). This work can help us understand the potential mechanism of chronic hepatitis B causing different disease outcomes and provide potential help for specific interventions.

## Alterations in Gut Microbiota Is Associated With Hepatitis B-Related Cirrhosis in Both Metabolic and Immune Ways

### Alterations of Gut Microbiota in Hepatitis B-Related Cirrhosis

With the development of multi-omics analysis, an increasing number of studies turned to uncovering the potential role of gut microbiota in human diseases and the underlying mechanisms. As a chronic liver disease, hepatitis B-related cirrhosis has also been linked to altered gut microbiota. By testing the gut microbiota of healthy people, HBV carriers, patients with chronic hepatitis B and patients with hepatitis B-related cirrhosis, Lu et al. found that the abundance of two opportunistic pathogens, *Enterococcus faecalis* and *Enterobacteriaceae* increased significantly, while the number of intestinal commensal bacteria, such as *Bifidobacterium*, *Faecalibacterium prausnitzii* and lactic acid bacteria (*Lactobacillus*, *Pediococcus*, *Leuconostoc*, and *Weissella*) decreased significantly in cirrhotic patients compared to the first three groups (Lu et al., 2011). *F. prausnitzii* is an anti-inflammatory bacterium that stimulates the secretion of interleukin (IL)-10 and inhibits the expression of IL-12 and interferon-gamma (IFN-γ) (Sokol et al., 2008), and butyrate has been identified as the medium (Lenoir et al., 2020). Wei et al. (Wei et al., 2013) applied a metabolomic approach to further analyze the structural and functional metabolic changes of gut microbiota in patients with liver cirrhosis and found that, in terms of the composition, the gut microbiota of patients with liver cirrhosis contained lower levels of Bacillariophyceae and higher levels of *Enterobacteriaceae*, *Veronococcaceae* and *Streptococcaceae* compared to the healthy group. In terms of functional metabolism, genes and proteins associated with the transport of executive substances (mainly amino acids and carbohydrates) were enriched in the gut microbiota of patients with cirrhosis, indicating that the metabolic activity of the gut microbiota increased in cirrhosis, which is consistent with the results of Chen et al. (Chen et al., 2014). This is due to the altered intestinal microenvironment in cirrhosis, which inhibits the growth of bacteria in the gut. These unfavorable conditions lead to an enhancement in the transport and metabolism of substances of some bacteria, creating more extensive metabolic pathways. In addition, Wei et al. found a significant impairment of bile acid metabolism in patients with hepatitis B-related cirrhosis compared to the healthy group (Wei et al., 2013). It is well known that there are mutual influences between bile acid metabolism and the gut microbiota. The primary bile acids delivered to the intestine can only be reabsorbed by the liver after being transformed into secondary bile acids by the dissociation and dehydroxylation of bile salt hydrolases produced by *Lactobacillus* and *Bifidobacterium*, etc. (Liang et al., 2018), while secondary bile acids (mainly deoxycholic acid) produced by dehydroxylation can also maintain the stability of the intrinsic gut microbiota by enhancing the barrier function of the intestinal mucosa and inhibiting the adhesion and colonization of harmful bacteria. In hepatitis B-related cirrhosis, disturbances in the gut microbiota lead to impaired bile acid dissociation and dehydroxylation, and consequently to disorders in bile acid metabolism. In return, the impaired transformation of BAs was also linked to altered gut microenvironment and microbiota. Oral commensal bacteria like *Streptococcus salivarius* (Zhang et al., 2013) and certain species belonging to *Veillonella* (Chen et al., 2016) were increased in gut microbiota in patients with liver cirrhosis, and some of them were able to produce ammonia, which may lead to hyperammonemia and even hepatic encephalopathy (Qin et al., 2014). The alterations in the quantity, structure and function of the gut microbiota in patients with hepatitis B-related cirrhosis also suggest that interventions on the gut microbiota might help improve the primary disease. Several randomized controlled trials evaluating the efficacy of probiotic treatment in patients with mild hepatic encephalopathy found that probiotic treatment significantly reduced serum endotoxin and ammonia levels and improved mild cognitive impairment compared to the placebo group (Dai et al., 2014; Dhiman et al., 2014; Xia et al., 2018). It has also been suggested that intestinal dysbiosis is involved in the development of spontaneous bacterial peritonitis (Oikonomou et al., 2018). Thus, it is clear that dysbiosis of gut microbiota is not only involved in the initiation and development of hepatitis B-related cirrhosis, but also promotes the development of related complications.

### Gut-Liver Axis and the Innate Immune Response of Liver Play Direct Roles in Local and Systemic Pro-Inflammatory Status

In hepatitis B-related cirrhosis, damage to hepatocytes arises not only from the cellular immune response caused by HBV infection, but also from the intrinsic immune response caused by pathogen-associated molecular patterns (PAMPs) produced by intestinal microorganisms. Pijls et al. showed that increased intestinal permeability in patients with compensated cirrhosis and the presence of a variety of large numbers of bacteria in the intestine may increase the risk of bacterial translocation (Pijls et al., 2014). During bacterial translocation, bacteria and their components like endotoxins, peptidoglycan and bacterial DNA translocate from the enteric cavity to mesenteric lymph nodes and other organs (Fukui, 2015). TLRs and NOD-like receptors (NLRs) are essential pattern recognition receptors (PRRs). They are expressed on the surface of Kupffer cells, hepatocytes, and plasmacytoid dendritic cells (pDCs) in the liver and peripheral blood mononuclear cells (PBMCs) in the circulation, playing a critical role in innate immunity. Gut microbiota dysbiosis causes Toll-like (TLR) and NOD-like (NLR) receptors activation in the liver. TLR/NLR further induced the host-wide inflammatory response by inducing the nuclear factor kappa B (NF-κB) transcriptional pathways, and accelerating the secretion of cytokines, such as tumor necrosis factor-alpha (TNF-α) (Chassaing et al., 2014). Due to the gut-liver axis, the liver is the first organ exposed to LPS from the gut. Though adaptive immunity is thought to be more important than innate immunity, chronic inflammation caused by LPS resulting from gut leakage is associated with the activation of liver injury. For instance, the main component of Gram-negative bacterial outer membrane, lipopolysaccharide (LPS), is transferred by CD14 to TLR4 (Kitchens and Thompson, 2005). Then TLR4/LPS complex activates NF-κB, and induces inflammatory cytokine production (Miyake, 2006), further causing injury and liver inflammation (Ben-Ari et al., 2012). Interestingly, TLR5, which recognizes flagellin, has been reported to mediate the crosstalk between innate immune and metabolic disorders like decreased sensitivity to insulin (Li et al., 2017). The negative effect of the gut microbiota dysbiosis on liver injury and inflammation influences the course of HBV disease (Lu et al., 2011). This prolonged and sustained inflammatory stimulus causes repeated damage to hepatocytes followed by regenerative repair, and the hepatic stellate cells (HSCs) are activated and trans-differentiated to mediate the initiation and development of fibrosis. These together exacerbate the histopathological damage of cirrhosis, and lead to various complications (Arab et al., 2018).

### Metabolism Regulated by Gut Microbiota Has Both Direct and Indirect Effects on Cirrhosis and Other Chronic Liver Diseases Related to Hepatitis B

The relationship between the dysbiosis of gut microbiota and hepatitis B has received increasing attention, and serum metabolites derived from or influenced by gut microbiota have been revealed to link the dysbiosis of microbiota and the progress or prognosis of hepatitis B. In a study, researchers enlisted 85 patients with chronic hepatitis B and 22 healthy volunteers matched in age, gender and body mass index, and compared their blood and fecal samples. It was founded that the gut microbiota in patients with chronic hepatitis B has already changed before the severe liver lesions and the shift in gut microbiota may be pathogenic in liver diseases (Niu et al., 2015). Gut microbiota was involved in the abnormal accumulation of serum metabolites, which was closely associated with liver disease (Schnabl and Brenner, 2014).

**Figure 2:**
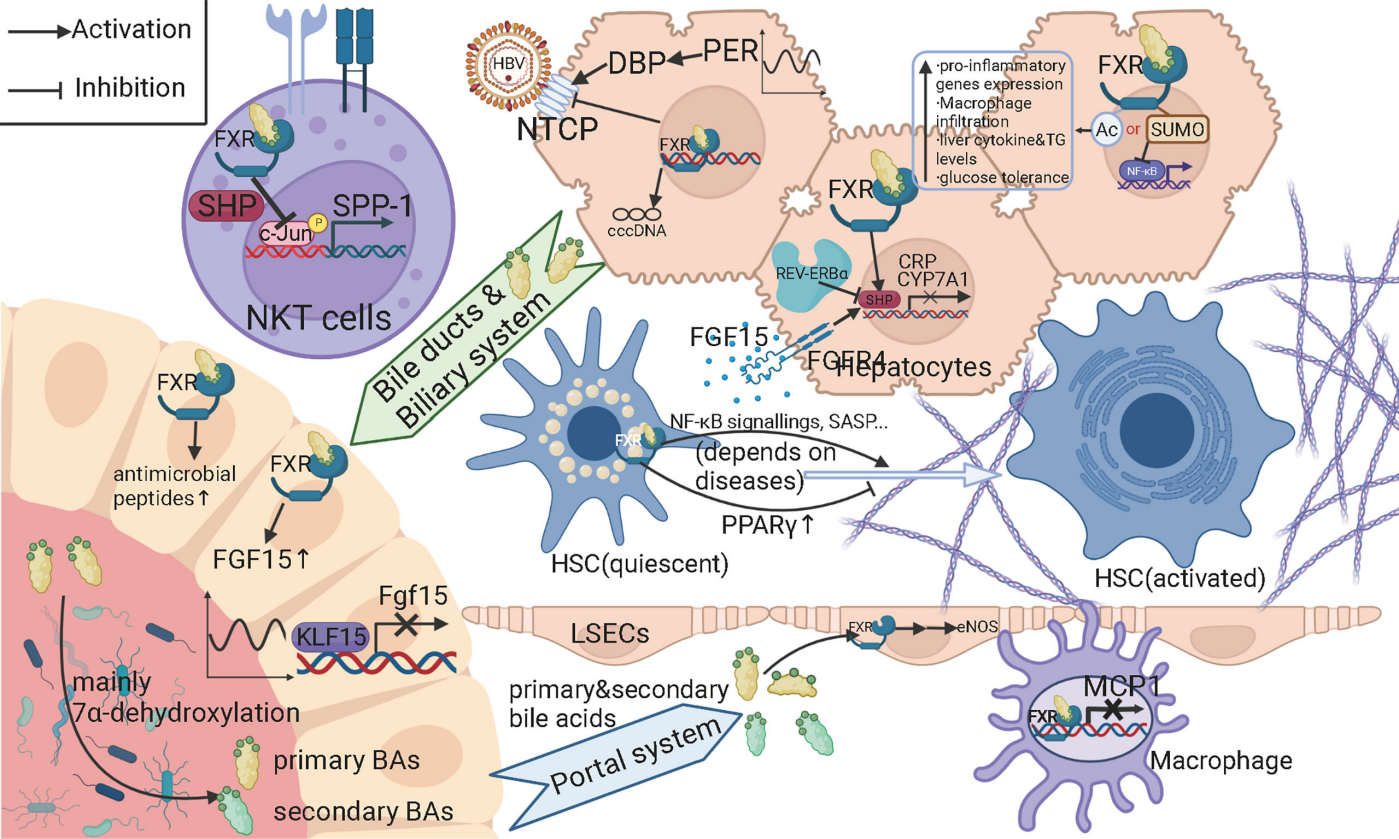
Schematic figure for the molecular mechanisms of bile acids and farnesoid X receptors (FXR) among gut microbiota, hepatitis B-related cirrhosis and circadian rhythms. As the speed-limiting enzyme in the classic pathway of bile acid synthesis, CYP7A1 can be downregulated by FXR activated by bile acids and fibroblast growth factor (FGF) receptor (FGFR) 4 activated by FGF15. FGF15 is expressed and secreted by intestinal endothelial cells (IECs). FXR activated by bile acids in IECs contributes to the expression of FGF15. Kruppel-like factor 15 (KLF15) give diurnal rhythms to the generation of FGF15. Partial primary bile acids are transformed into secondary bile acids mainly through the 7alpha-dehydroxylation of certain members of gut microbiota. FXR binding with bile acids can upregulate antimicrobial peptides, which can suppress the overgrowth of gut microbiota. Primary and secondary bile acids return to liver *via* portal system. In liver sinusoidal endothelial cells (LSECs), bile acids can activate the FXR and leading to the upregulation of endothelial nitric oxide synthase (eNOS), increasing the level of NO and decrease the pressure in portal system. Secondary bile acids can activate NF-κB signaling and senescence-associated secretary phenotype (SASP) in hepatic stellate cells (HSCs), thus promoting cirrhosis, while under some circumstances, bile acids can prevent the activation of HSCs by upregulating peroxisome proliferation-associated receptor gamma (PPARγ). FXR binding with bile acids can inhibit the expression of osteopontin in natural killer T (NKT) cells, the interleukin (IL)-6 induced expression of C reactive protein (CRP) in hepatocytes, and the expression of monocyte chemoattractant protein-1 (MCP1) in macrophages. Acetylation of FXR leads to a series of pro-inflammatory issues and impaired substance metabolism, while small ubiquitin-like modifier (SUMO)-ylation of FXR can inhibit the expression of NF-κB pathway-related molecules. FXR can also downregulate the expression of Na+-taurocholate co-transporting polypeptide (NTCP) and perturb the infection of hepatitis B virus (HBV). The transcription of HBV is also regulated differently when FXR is activated by different ligands. Period (Per) and REV-ERBα, as central circadian clock transcription factors, upregulates NTCP and CYP7A1, respectively.

As mentioned above, bile acids (BAs) and their metabolism can be essential and characteristic in their impact on hepatitis B-related cirrhosis since BAs mainly circulates between the gut and liver. In addition to assisting in the absorption of lipids, fat-soluble drugs, and vitamins in the small intestine, BAs also act as important signaling molecules involved in energy and substance metabolism processes. Hepatocytes use cholesterol as a raw material for the synthesis of primary bile acids such as bile acid (CA) and goose deoxycholic acid (CDCA) *via* the classical and bypass pathways. After combining with glycine or taurine, primary bile acids enter the intestine *via* the bile ducts, most of which are reabsorbed in the ileum into the portal vein to the liver, forming the enterohepatic circulation, while a small proportion is converted into secondary bile acids such as lithocholic acid (LCA) and deoxycholic acid (DCA) by the action of gut microbiota (Jia et al., 2018). Colonic 7α-dehydroxylating bacteria (e.g. *Lachnospiraceae*, *Ruminococcaceae*, and *Blautia*) play a key role in this transformation process. On the other hand, BAs have antimicrobial properties and can affect the composition and structure of the gut microbiota, either directly or indirectly through the synthesis of antimicrobial peptides (Ridlon et al., 2015). The interaction between bile acids and gut microbiota is therefore important for the maintenance of homeostasis in the body. Once the homeostasis is imbalanced, an inflammatory response is induced, which can lead to chronic inflammation of the liver and liver fibrosis, and even finally to cirrhosis and liver cancer. The results of Kakiyama et al. (Kakiyama et al., 2013) showed that patients with cirrhosis had a higher abundance of *Enterobacteriaceae* bacteria and a lower abundance of *Lachnospiraceae*, *Ruminococcaceae*, and *Blautia* compared to controls; CDCA was positively correlated with *Enterobacteriaceae*, while DCA was positively correlated with *Ruminococcaceae*. These results suggest that reduced conversion of primary to secondary BAs is associated with the gut microbiota of patients with decompensated cirrhosis. Their results are consistent with those of Wang et al. (Wang et al., 2020), which demonstrated that patients with chronic hepatitis B have lower levels of secondary BAs and higher levels of primary BAs. Dysbiosis of the gut microbiota can indirectly lead to inflammation by affecting the production of secondary BAs, so theoretically, regulating the intestinal flora could delay the development of cirrhosis and its complications. BAs bind to the farnesoid X receptor (FXR) in hepatocytes and intestinal epithelial cells and to the G protein-coupled bile acid receptor (GP-BAR1, also named TGR5) in hepatic nonparenchymal cells to activate various signaling pathways that regulate multiple metabolic processes such as the metabolism of triglyceride, cholesterol, glucose and inflammatory responses (Ridlon et al., 2015). FXR has been shown to have the capacity for alleviating liver inflammation *via* various signaling pathways. Inducing PPARγ (Fiorucci et al., 2005), IL-6-induced C-reactive protein (CRP) (Zhang et al., 2009), c-Jun-promoted osteopontin expression and secretion in NKT cells (Mencarelli et al., 2009), monocyte chemoattractant protein-1 (MCP-1) (Li et al., 2015), and acetylation or small ubiquitin-like modifier (SUMO)-ylation of FXR (Kim et al., 2015) are all targets or manners through which activated FXR reduced the level of liver inflammation. (Verbeke et al., 2014) found that the FXR agonist obeticholic acid reduced portal pressure in a rat model of cirrhosis by decreasing intrahepatic vascular resistance through increased endothelial nitric oxide synthase (eNOS) activity in the liver. Besides FXR, the activation of TGR5 highly expressed on the surface of liver sinusoidal endothelial cells (LSECs) also leads to the upregulation of eNOS in LSECs, which is able to attenuate portal hypertension caused by cirrhosis (Keitel et al., 2007). In the gut, the activation of FXR induces the expression of antimicrobial peptides, which can prevent the overgrowth of gut bacteria (Sun et al., 2021a). FXR agonists also prevented intestinal barrier dysfunction, intestinal inflammation and bacterial translocation in cholestatic rats. However, under certain situations, the binding of bile acids and FXR might serve as a pro-cirrhosis factor. The results of Saga et al. (Saga et al., 2018) pointed out that secondary unconjugated BAs could lead to the higher expression of genes related to NF-κB pathway, with the level of secreted IL-6 increasing. At the same time, the expression of α-Smooth actin (α-SMA), a marker of the activated HSCs, was significantly upregulated in HSCs treated with secondary unconjugated BAs. Interestingly, senescence-associated secretory phenotypes were also observed after secondary unconjugated BAs treatment. Garrido et al. (Garrido et al., 2022) showed that the accumulation of bile acids in liver sinusoids, which was resulted from the histone acetylation of the gene encoding Na^+^-taurocholate co-transporting polypeptide (NTCP), led to the activation of HSCs through FXR and progression of cirrhosis. A study conducted by Michel et al. (Fausther and Dranoff, 2011) showed that FXR loss selectively reduced the biliary cirrhosis and exerted no effects on non-cholestatic cirrhosis like CCl_4_-intoxication- and *Schistosoma mansoni*-induced cirrhosis. All these results suggested that FXR is a multi-functional receptor of bile acids with different signaling pathways and effects under different disease states, with different modifications, or when expressed in different types of cells. For hepatitis B, the impacts of FXR signaling appear to be stage-specific. FXRα activated by bile acids, a subtype of FXR, might lead to the enhanced transcription of HBV, while FXRα activated by synthetic agonists reversely reduced the level of HBV-related nucleic acids and proteins. Complete or partial silencing of FXRα gene led to the reduction of different HBV components, indicating that there were multiple underlying mechanisms (Radreau et al., 2016; Mouzannar et al., 2019). A dual agonist of FXR and TGR5 demonstrated the ability to perturb the infection of HBV, making it a promising candidate for new anti-HBV drugs (Ito et al., 2021)(**Figure 2**). The role of FXR in the progression of hepatitis B-related cirrhosis still require further research to achieve a more comprehensive understanding of the function of FXR in cirrhosis and provide new therapeutic targets in the treatment of hepatitis B-related cirrhosis.

In addition to impacting the progress of chronic hepatitis B-related cirrhosis directly, metabolites derived from or regulated by gut microbiota also serve as cues of innate and adaptive immunity. Short-chain fatty acids (SCFAs) have been identified to have a positive immunomodulatory effect. Intestine IL-22 produced by innate lymphoid cells (ILCs) and CD4+ T cells is important for local immunity in the intestine. SCFAs have been shown to be essential for gut microbiota to stimulate the secretion of IL-22 through an epigenetic way (Yang et al., 2020). As for the anti-tumor immunity, cancer patients with higher serum butyrate showed a higher sensitivity to the treatment of oxaliplatin. *In vitro* and *in vivo* experiments both indicated that butyrate enhanced the function of CD8+ cytotoxic T cells in an IL-12-ID2-dependent way (He et al., 2021). Furthermore, SCFAs treatment was demonstrated to be able to delay the onset of hepatocyte cancer (HCC) from chronic liver diseases (CLDs) in HBx transgenic mice (McBrearty et al., 2021). This result indicated that SCFAs treatment may have the potential to improve the prognosis and lead to better clinical outcomes through its anti-neoplastic properties. Butyrate, which has been reported to promote the extrathymic generation of regulatory T cells and thus to exhibit an anti-inflammatory property (Arpaia et al., 2013), was demonstrated to induce B10 cells to produce IL-10, an anti-inflammatory cytokine, through its regulation of retinoic acids receptor (RAR)-related orphan receptor alpha (RORα)-NR1D1 pathway. Mice experiments supported that this bioactivity of butyrate could be applied to the treatment of Sjogren’s syndrome, an autoimmune disease damaging exocrine glands like salivary glands (Kim et al., 2021). Besides immunomodulatory properties, the SCFAs also have an essential capacity of regulating metabolism. SCFAs have been reported to reduce fat-storage (Adolph et al., 2018; Stephens et al., 2018) in liver and to improve the homeostasis of lipid metabolism at multiple levels, varying from the production and secretion of insulin (Boursier et al., 2016) and other endocrine hormones (Tolhurst et al., 2012) to the balance between lipogenesis and lipolysis (den Besten et al., 2015).

## Interactions Between Circadian Rhythms and Gut Microbiota Exert Considerable Influence on Homeostasis: The Potential Mechanism

In view of the important role of gut microbiota in chronic hepatitis B and the close relationship between microbiota and host rhythm, we further discussed the interaction and molecular mechanism of the latter two. This part could offer valuable clues and candidates involved in the interactions between circadian rhythms and gut microbiota to imply their potential mechanism for chronic hepatitis B.

### Circadian Clock Affects Gut Microbiota Mainly Through Diet, Sleep, and Light/Dark Cycles

The circadian rhythm system of humans is a hierarchical system. The central circadian oscillator locates in the suprachiasmatic nucleus of the anterior hypothalamus, which in turn synchronizes peripheral circadian, thus dictating the expression of a set of core clock genes in every cell mainly including clock, bmal1, per1/2/3, and cry1/2 (Froy, 2011; Chen and Yang, 2014). Light/dark cycles are the most potent but not the only cue that controls the circadian rhythm. Diet and sleep also participate in the regulation of circadian rhythm (Parkar et al., 2019) while being modulated by light/dark cycles.

Many physiological processes, including digestion, metabolism, and the function of the immune system, are under the regulation of the symbiont gut microbiota, which serves as an acquired organ (Sommer and Backhed, 2013). Current studies demonstrated that gut microbial dysbiosis may result from circadian misalignment, indicating the trans-kingdom influence of human circadian clock (Thaiss et al., 2014). Diet and sleep that may disturb the host circadian system also influent the gut microbiota (Parkar et al., 2019).

Diet has a crucial impact on gut microbiota as it directly supplies energy to intestinal bacteria. A study published in Nature in 2014 demonstrated that the abundance of the gut microbiome could rapidly respond to an altered diet (David et al., 2014). And long-term changes in diet have also been demonstrated to alter the population of bacterial species in the gut (Wu et al., 2011). Feeding time influences the daily structural fluctuations and the quantitative oscillations of the gut microbiota (Zarrinpar et al., 2016). Ablation of clock genes reduces the eating rhythmicity of host. Christoph A. Thaiss’ group observed that Per1/2-/- mice almost completely lost the diurnal variations of gut microbiota (Thaiss et al., 2014).

Sleep disorders cause a significant effect on circadian rhythm disruption, which are associated with metabolic changes and contribute to health issues (Archer and Oster, 2015). The relevance between gut microbiota and sleep disturbance has been discussed in recent years. A randomized crossover study demonstrated that short-term sleep deprivation can induce subtle effects on human gut microbiota (Benedict et al., 2016). In a study published in 2016, mice were exposed to sleep fragmentation for 4 weeks and then allowed to recover for 2 weeks. Then tissues inflammation, representing an imbalanced immunity, and disturbance of metabolic homeostasis mediated by alterations in gut microbiota were reported. They demonstrated that chronic sleep disruption led to selective alterations in gut microbiota that elicited concurrent systemic inflammatory changes (Dobnik et al., 2016). Additionally, hypertension induced by Obstructive Sleep Apnoea (OSA) influences the high-fat diet-induced gut dysbiosis (Durgan et al., 2016). Poor sleep quality altered gut microbiota composition resulting in lower cognitive flexibility (Anderson et al., 2017).

Mice living in normal light/dark cycles eat mainly in the nocturnal phase, resulting in an increase of microbial pathways related to energy metabolism, DNA repair, and cell growth within the same phase with or without a delay. Contrarily, resting in the light phase led to an increase in microbial pathways related to chemotaxis and motility required for the mucus-adherent bacteria to reach closer to the intestinal wall (Liang et al., 2015; Thaiss et al., 2016). Feeding restricted to the active dark phase resulted in fluctuations of microbial abundance of the mouse gut microbiota, with *Firmicutes* peaking during feeding and decreasing during daytime fasting with a peak-to-trough ratio of 3:1. *Bacteroidetes* and *Verrucomicrobia* peaked during the daytime fasting period (Zarrinpar et al., 2014). Different studies on mice indicated that the peaks in *Firmicutes* during the dark phase are diet-driven, while light blooms in *Bacteroidetes* and *Verrucomicrobia* during the light phase were due to the cessation of feeding (Parkar et al., 2019). Thus, circadian clock could help understand the potential mechanism of gut microbiota involved in the diverse outcome of chronic hepatitis B.

### Bile Acids Metabolism Might Serve as a Potential Link Between Circadian Rhythms and Gut Microbiota

Bile acids are important for the ingestion of lipids and its discharge from gallbladder is closely associated with diet, so it has early been observed that there is a rhythmic oscillation in bile acids concentrations in the digestive tract and systemic circulation and the peak comes after the food intake (Setchell et al., 1982). Since mice have a nocturnal feeding habit, the highest concentrations of bile acids in serum and intestinal lumen appear at night (Han et al., 2015). The rhythmic expression of speed-limiting enzymes in both classic and bypass pathways of bile acids biosynthesis is the central part of the diurnal rhythms in bile acids metabolism. Studies on mice which have more regular and controllable feeding habits have provided solid evidences. *Cyp7a1*, encoding the speed-limiting enzyme CYP7A1 in the classic pathway of bile acids synthesis, has an increasing expression starting from daytime and peak after eating in a dark time. In humans, 1 p.m. and 9 p.m. are the two-timing when bile acids synthesis is most active (Axelson et al., 1988; Galman et al., 2005). Genes in the bypass pathway share a similar pattern of rhythm with Cyp7a1 (Wu et al., 2015). Besides nonphotic Zeitgeber like feeding, circadian clock genes also play a role in regulating the rhythm of bile acids metabolism. REV-ERBα (also known as NR1D1, nuclear receptor superfamily 1 group D number1), a central molecular in the cell-intrinsic circadian clock, downregulates SHP (small heterodimer partner) and E4BP4 to promote the expression of *Cyp7a1* (Duez et al., 2008; Le Martelot et al., 2009). On the other side of enterohepatic circulation of bile acids, FXR in intestinal epithelial cells (IECs) binding to and activated by bile acids induce the expression of fibroblast growth factor 15 (Fgf15) which is able to downregulate CYP7A1 in hepatocytes through FGF15-FGR4 signaling axis (Inagaki et al., 2005; Song et al., 2009). FGF15 expression in IECs is also negatively regulated by a transcription factor with diurnally rhythmic expression called Kruppel-like factor 15 (KLF15) (Han et al., 2015). Transporters in the enterohepatic circulation of bile acids are also targets for regulations. Per (period) has been showed to be essential for the rhythmic expression of NTCP on the surface of hepatocytes (Ma et al., 2009)(**Figure 2**). Given that bile acids also demonstrate the capacity for modulating gut microbiota, there are also growing studies focusing on how bile acids metabolism links circadian rhythms to gut microbiota. A recent study conducted by Cui et al. (Cui et al., 2022) reported that apple polyphenol extract (APE) could induce the daily rhythm of circadian genes expression in SCN and ileum in mice fed with high-fat diet (HFD). Gut microbiota, rhythms of FXR expression in liver and ileum, and bile acids profile were also significantly altered by the administration of APE. Emerging pieces of evidence are suggesting that the triangle network of circadian rhythms, bile acids metabolism, and gut microbiota might provide new perspectives for research, especially on metabolic and systemic diseases.

### Gut Microbiota Regulates the Expression of Circadian Clock-Related Genes and Affects Metabolism and Immunity Sequentially

The circadian clock intrinsic to every cell is actually a group of transcription-translation loops. Among these feedback loops, two loops are in the leading position. One consists of CLOCK (circadian locomotor output cycles kaput), BMAL1(also known as ARNTL, aryl hydrocarbon receptor nuclear translocator-like protein 1), PER, and CRY (cryptochrome), among which the former two are promotive ones and the latter two can suppress the former two, and thus form a negative feedback loop and generate a diurnal rhythm. The other one includes REV-ERBα and retinoic acids receptor (RAR)-related orphan receptor family like RORα and RORγt. DBP (D site binding protein) and PPARGC1A (peroxisome proliferator-activated receptor gamma (PPARγ) co-activator 1α) are among the genes regulated by the second circadian clock loop. Many of these genes related to cell-intrinsic circadian clock and genes governed by them are essential in energy balance and immunity (Woldt et al., 2013; Wang et al., 2017; Brooks et al., 2021). The signaling relay consists of gut microbiota, TLR-MyD88-IL-23 pathway in DCs, IL-22 secretion of innate lymphoid cells group 3(ILC3) after being stimulated by IL-23, and IL-22-IL-22R-STAT3 phosphorylation pathway in intestine epithelial cells(IECs) has been shown to regulate the transport of lipids (Wang et al., 2017) and the secretion of antimicrobial peptides (Brooks et al., 2021). These results suggested both immunomodulatory and metabolic roles of circadian rhythms in gut microbiota-host interaction. Besides affecting the local diurnal rhythm, gut microbiota has been reported to affect the cell-intrinsic circadian clock in distant organs like the liver. A high-fat diet induces the changes in gut microbiota and the alterations in gut microbiota activate the PPARγ-mediated transcriptional reprogramming in hepatocytes, including the metabolic phenotype. The observation of increased long-chain fatty acids in signaling pathways and liver lipid accumulation was consistent with the conclusion (Murakami et al., 2016). Therefore, due to the linage of circadian rhythms between the gut microbiota and the host metabolism and immune status, circadian clock-related genes could be targeted as the candidates for understanding the mechanism of and intervention for chronic hepatitis B, including hepatitis B-related cirrhosis.

## Conclusions and Perspectives

As discussed above, the changes in living habits influence the abundance and rhythmicity of gut microbiota, leading to gut microbiota dysbiosis. Besides light/dark cycles, diet, and sleep, other living habits about circadian rhythms are also relevant to gut microbiota, such as jet lag (Cui et al., 2016; Parkar et al., 2019), the results of which will help explain the diverse effects of circadian rhythms on gut microbiota further. The development of microbiomics and multi-omics analysis has thrown new insights into the mechanisms and potential targets for treatment of hepatitis B-related cirrhosis. Circadian rhythm is among these variables and has been revealed to have a strong interaction with gut microbiota. Recently, inhibiting CutC, a bacterial gene encoding choline TMA(trimethylamine)-lyase, was shown to alter the host circadian regulation of phosphatidylcholine and energy metabolism, which consequently improved obesity induced by high fat diet or lack of leptin (Schugar et al., 2022). As the gut microbiota may change in HBV patients before the severe liver lesions and may be correlated to certain indexes like the hepatitis B viral load (Joo et al., 2021), the predicted value of gut microbiota dysbiosis to early diagnosis of hepatitis B-related cirrhosis is worthy of research. Interestingly, it was reported that a circadian gene dbp (encoding d site binding protein, DBP) was upregulated in HBsAg transgenic mice. The baseline abundance and amplitude of upregulation of dbp is larger in female mice, compared with those in male mice (Li et al., 2008). Though there were few studies on the clinical significance of dbp and its change in HBV infection, the results still provided evidence that HBV infection might modulate the peripheral circadian clock in infected hepatocytes and circadian clock-related genes might also be a target for developing more effective therapy. An integrated study based on patients with liver fibrosis of different severity showed that probiotics like *Lactobacillus* decreased in all fibrosis groups while pathogens increased. As the fibrosis progresses, unconjugated BAs in feces increased and conjugated BAs in serum went the opposite. In fibrosis groups, the FXR-SHP signaling pathway was downregulated in liver and ileum (Xiang et al., 2022).

In this review, we concluded that gut microbiota dysbiosis may be involved in abnormal accumulation of serum metabolites (Sun et al., 2021b), disruption of intestinal barrier and induction of TLR/NLR-pathway inflammatory reaction, which can influence the outcome of chronic hepatitis B, including the progression of cirrhosis. And at the same time, circadian rhythms have the potential ability to regulate the alterations of gut microbiota to participate in the diagnosis, progress, and prognosis of HBV-related disease. Developing a time-based medication regimen would be a promising direction in which studies on circadian rhythms can focus. What should be improved and furtherly investigated is the directive evidence of the influence of gut microbiota-circadian rhythms interactions on chronic hepatitis B and cirrhosis.

As for the further understanding of diverse outcomes of hepatitis B-related cirrhosis and the development of individualized and time-based therapies, we proposed a few suggestions as followings:

1. Approaches of systems biology would help to integrate circadian rhythms, gut microbiota, and cirrhosis into a whole and to observe the alterations when one part is changed.
2. Gut microbiota-derived metabolites and their organ-specific or tissue-specific signaling pathways would be powerful candidates for etiological treatment of liver cirrhosis, and a both phenotype- and transcriptome-oriented screening method may be more sensitive in discovering therapeutical chemicals.
3. Due to the lack of satisfying and convenient indexes for fibrosis and cirrhosis screening, integrated serum metabonomics, fecal metabonomics, and gut mirobiomics analysis is necessary to develop biomarkers or combinations of several biomarkers with both high sensitivity and high specificity.
4. Adjusting the time pattern of drug ministration may serve as a promising improvement, especially for drugs derived from endogenous substances and drugs targeting signaling pathways with natural circadian oscillations. More related pre-clinical animal experiments and clinical trials are needed.

## Author Contributions

TW and CZ designed, searched and collected the literature and wrote the manuscript. XR and CZ supervised the overall investigations and helps in the manuscript editing. All authors contributed to the article and approved the submitted version.

## Funding

This work is supported by the Natural Science Foundation of Shanghai (21ZR1409200) and National Key Research and Development Project of China (2018YFC2000500/03). TW is supported by the Qingfeng Project from Shanghai Medical College, Fudan University (QF2114). CZ is supported by outstanding talent from Fudan University (2015).

## Conflict of Interest

The authors declare that the research was conducted in the absence of any commercial or financial relationships that could be construed as a potential conflict of interest.

## Publisher’s Note

All claims expressed in this article are solely those of the authors and do not necessarily represent those of their affiliated organizations, or those of the publisher, the editors and the reviewers. Any product that may be evaluated in this article, or claim that may be made by its manufacturer, is not guaranteed or endorsed by the publisher.
